# Association of *ACE* and *AGTR1* variants with retinopathy of prematurity: a case–control study and meta-analysis

**DOI:** 10.1007/s13353-024-00900-0

**Published:** 2024-08-26

**Authors:** Anna Durska, Dawid Szpecht, Anna Gotz-Więckowska, Ewa Strauss

**Affiliations:** 1 Institute of Human Genetics, Polish Academy of Sciences, Strzeszynska 32, 60-479 Poznan, Poland; 2 Department of Neonatology, Poznan University of Medical Sciences, Poznan, Poland; 3 Department of Ophthalmology, Poznan University of Medical Sciences, Poznan, Poland

**Keywords:** Retinopathy of prematurity, Angiotensin-converting enzyme, *ACE*, Angiotensin II receptor type 1, *AGTR1*

## Abstract

Retinopathy of prematurity (ROP) is a major cause of childhood blindness worldwide, linked to gene variants in the renin–angiotensin–aldosterone system, including angiotensin-converting enzyme (*ACE*) and angiotensin II receptor type 1 (*AGTR1*). This study aims to evaluate the association between *ACE* insertion/deletion (I/D) and *AGTR1* rs5186A > C variants with the occurrence and progression of ROP in a Polish cohort. A total of 377 premature infants were enrolled in the study. The *ACE* variant was evaluated using PCR, and *AGTR1* was assessed using TaqMan probes. Clinical characteristics, including risk factors and comorbidities, were documented. A meta-analysis of the effects of the studied variants on ROP was also conducted. The *AGTR1* rs5186C allele was significantly associated with both the progression of ROP and treatment outcomes. Homozygotes exhibited a 2.47-fold increased risk of developing proliferative ROP and a 4.82-fold increased risk of treatment failure. The impact of this allele increased at low birth weight. A meta-analysis, including 191 cases and 1661 controls, indicated an overall risk of 1.7 (95%CI 1.02–2.84) for the recessive effect of the rs5186C allele. The *ACE* variant did not show a significant association with ROP in our population; however, a meta-analysis of 996 cases and 2787 controls suggested a recessive effect of the insertion allele (an odds ratio of 1.21 (95%CI 1.00–1.60)). These results indicate that gain-of-function *AGTR1* variants may play a crucial role in the development of ROP, potentially by promoting angiogenesis and pro-inflammatory effects. Screening for these variants could facilitate the development of personalized risk assessment and treatment strategies for ROP.

**Supplementary Information:**

The online version contains supplementary material available at 10.1007/s13353-024-00900-0.

## Introduction

Retinopathy of prematurity (ROP) is a predominant cause of childhood blindness worldwide (Kassabian et al. 2020). The etiology of ROP is multifaceted, with birth weight (BW) and gestational age (GA) of the premature infant identified as principal risk factors (Broxterman and Hug 2016). Additionally, ROP has also been linked to various environmental factors, including excessive and uncontrolled supplemental oxygen exposure at birth as well as oxidative stress (Patz 1980; York et al. 2004). There is substantial evidence suggesting a strong genetic predisposition for ROP, as indicated by the variation in racial and regional prevalence of the ROP (Modrzejewska and Bosy-Gąsior 2023) and risk factors (Darlow et al. 2005) (Schaffer et al. 1993; Ng et al. 1988), as well as a high heritability rate, estimated to be as much as 70% (Bizzarro et al. 2006).

Candidate gene analyses have identified an association between gene variants encoding the renin–angiotensin–aldosterone system (RAAS) and ROP (Swan et al. 2018). RAAS is a complex network essential for ensuring perfusion of critical organs, contributes in proper vessels development, but also participates in pathological angiogenesis. Prior studies showed that the development of ROP may be influenced by variants in two genes encoding components of the RAAS: the angiotensin-converting enzyme gene (*ACE*) (Haider et al. 2002; Yildiz et al. 2010; Lei et al. 2018; Poggi et al. 2015; Spiegler et al. 2010) and the angiotensin II receptor type 1 gene (*AGTR1*) (Poggi et al. 2015; Spiegler et al. 2010; Rathi et al. 2017; Mohamed et al. 2009). ACE is active in hydrolyzing angiotensin I (Ang I) to angiotensin II (Ang II) (St Paul et al. 2020), which acts as a pleiotropic hormone affecting multiple processes, contributing to the development of cardiovascular disease and regulation of vascular tone (Vukelic and Griendling 2014). Angiotensin II interacts with two pharmacologically distinct subtypes of cell surface receptors: types 1 (AGTR1) and 2 (AGTR2). Type 1 receptors are the main effectors of the RAAS and mediate the cardiovascular effects of angiotensin II, while their activity is antagonized by type 2 receptors. The inhibition of the RAAS, using ACE inhibitors (ACEi) or angiotensin receptor blockers, has been shown to mitigate oxygen-induced retinopathy (Moravski et al. 2000; Shi et al. 2023), suggesting that RAAS inhibition could be a promising therapeutic approach for ROP. Among ACEi, enalaprilat has potential as a novel means of preventing ROP development (Katargina et al. 2023).

Despite initial promising insights into the role of RAAS genes in ROP, the currently available data remain limited. Previous genetic association studies are inconsistent and inconclusive, with no data available from Central European countries. This study aims to analyze the involvement of the *ACE* insertion/deletion (I/D of a 287-bp fragment) variant in intron 16 and *AGTR1* rs5186 single nucleotide variant (SNV, 1166A > C) in 3′UTR in a cohort of premature infants from the Polish population. Additionally, we conducted a meta-analysis of relevant studies to obtain a more comprehensive understanding of the role of these genes in the occurrence of ROP.

## Patients and methods

### Study population

This study included 377 Caucasian preterm infants born between 22 and < 32 weeks of gestation, who were hospitalized between 2009 and 2020 at the Gynecology and Obstetrics Clinical Hospital of Poznan University of Medical Sciences. Exclusion criteria included neonates from multiple pregnancies, those with chromosomal abnormalities or TORCH infections, and infants who did not receive antenatal steroid therapy.

### Clinical characteristics and ROP management

Clinical characteristics, including GA (weeks), BW (grams), sex, Apgar scores at 1 and 5 min, and parameters related to respiratory failure (such as use of surfactant, resuscitation, duration of mechanical ventilation), were reported. The criteria for diagnosing extremely low GA (< 28 weeks, ELGA) and extremely low BW (< 1000 g, ELBW) were established based on WHO guidelines (WHO 2022). The premature newborns included in the study underwent regular ROP screening. The initial examination occurred in the 4th week of chronological age, followed by subsequent exams every 7–10 days, depending on eye condition. When ROP was diagnosed, fundus lesions were classified per the International Classification of Retinopathy of Prematurity. Treatment indications followed the Early Treatment for Retinopathy of Prematurity guidelines. Options included laser photocoagulation of the peripheral avascular retina or intravitreal anti-VEGF antibody (ranibizumab) administration within 72 h of diagnosis. ROP screening continued until vascularization reached zone III or until signs of ROP regression were observed in at least two consecutive exams. Treatment failure was indicated by the absence of anatomical ROP regression markers following treatment. Similarly sized groups of children without ROP, children with spontaneously regressed ROP, and those with ROP requiring treatment were recruited simultaneously mainly during ophthalmic screening studies, considering the limitations related to the number of children treated. The observed frequencies of cases in the presented case–control study do not correspond to those in the general population of premature infants. In addition to ROP, data on the incidence of several comorbidities associated with prematurity were collected, including respiratory distress syndrome (RDS), intraventricular hemorrhage (IVH), diffuse white matter injury (DWMI), necrotizing enterocolitis (NEC), and bronchopulmonary dysplasia (BPD).

### Genotyping

Genomic DNA was extracted from buccal swabs using the innuPREP DNA Kit (Analytik Jena AG, Jena, Germany) or from circulating blood lymphocytes using the QIAamp DNA Kit (Qiagen GmbH, Hilden, Germany), according to the manufacturer’s protocols. Genotyping of the *ACE* I/D variation was performed by two separate PCR reactions. The first reaction identified both alleles (insertion and deletion) using a primer pair described by Rigat et al. (1992). The second reaction specifically identified the I allele using a primer pair described by Lindpaintner et al. (1995). Due to the preferential amplification of the D allele in heterozygous samples, each sample identified as DD genotype in the initial step underwent a secondary PCR reaction with primers targeting an insertion-specific sequence. This secondary reaction produced a 335-bp amplicon exclusively in the presence of the I allele, with no product in samples homozygous for DD. Primers were obtained from Genomed. The presence of the *AGTR1* rs5186 variant was determined using a predesigned TaqMan SNP genotyping assay (test no. C___3187716_10; Thermo Fisher Scientific, Waltham, MA, USA), on the ABI 7900HT Fast Real-Time PCR System (Life Technologies, Carlsbad, California). Details of the methodology for evaluating the studied variants are presented in Supplementary Table S1. The genotyping success rate were 99.7% for *ACE* and 99.1% for *AGTR1* variants.

### Ethical statement

All procedures carried out on human participants in this study were in accordance with the ethical standards of the institutional and/or national research committee and with the 1964 Helsinki declaration and its later amendments (or comparable ethical standards). The study was approved by the Bioethics Committee of Poznan University of Medical Sciences (no. 1140/05, 1117/18). Written prior-informed consent was obtained from the parents or guardians of the patients.

### Meta-analysis *ACE* and *AGTR1* variants and ROP

The meta-analysis was conducted following the Preferred Reporting Items for Systematic Reviews and Meta-Analyses (PRISMA) statement (Moher et al. 2009). Associations with ROP development and progression were studied separately.

- Search strategy
- A literature search was conducted in PubMed, Embase, Web of Science, and Scopus to identify relevant studies published in English on the association of the studied ACE and AGTR1 variants with ROP. The search was performed up until November 01, 2023. Various combinations of keywords were used: (a) “angiotensin-converting enzyme” or “ACE,” “polymorphism” or “mutation” or “variant” or “I/D” or “ID” or “rs4646994,” “angiotensin II receptor type 1” or “ACE,” “polymorphism” or “mutation” or “variant” or “I/D” or “rs4646994,” (b) “angiotensin II receptor type 1” or “AGTR1” or “AT2R1” or “AT1R” or “polymorphism” or “mutation” or “variant” or “rs5186,” and “retinopathy of prematurity” or “ROP.” Additionally, the reference lists of retrieved articles and previous reviews were manually searched to ensure the inclusion of all relevant studies.
- Inclusion and exclusion criteria
- The following criteria were applied to include studies in the meta-analysis: (a) studies with a case–control or cohort design; (b) studies focusing on the association between ACE or AGTR1 variants and ROP; (c) availability of sufficient genotype data in the case and control groups to calculate crude ORs and 95% CIs. The exclusion criteria were as follows: (a) studies not designed as case–control or cohort studies; (b) the lack of genotype data or inability to calculate it; (c) studies based on pedigree data, twins studies, linkage and family-based studies; (d) case reports, review articles, posters, abstracts, and animal studies; and (e) studies with incomplete or overlapping data. In cases of overlapping or duplicate publications only the largest or most recently updated sample data was included.
- Data extraction
- Two authors independently reviewed and extracted the following information from all included studies using a structured data collection: first author name, publication date, genotyping method, participant location, ethnicity, genotyping methods, sample sizes of cases and controls, genotype frequency distribution, minor allele frequency (MAF), and Hardy–Weinberg equilibrium (HWE) in controls. Any discrepancies were resolved through consensus among all authors. If the included studies did not provide detailed genotype or HWE information, we calculated them and provided the relevant information.
- Quality assessment
- The quality of studies was independently assessed by the Newcastle–Ottawa quality scale (NOS) (Wells et al. 2017; Luchini et al. 2017; 2021). Studies with scores of 0–3, 4–6, and 7–9 were, respectively, considered as low, moderate, and high quality.

### Statistical analysis

For continuous parameters, deviations from the normal distribution were assessed using the Kolmogorov–Smirnov test. To evaluate the individual effects of the studied clinical risk factors on the onset and progression of ROP, a linear regression analysis or Spearman rank order correlation was used, with *P-*values for the trends calculated. In these analyses, patients groups were categorized as follows: 0, no ROP; 1, ROP requiring treatment; and 2, ROP not requiring treatment. For other univariate analyses comparing two groups, qualitative variables were examined using the *χ*^*2*^ test or Fisher’s test, while quantitative variables were evaluated using the *t*-test or Mann–Whitney *U* test. Odds ratios (ORs) with 95% CIs were calculated for the genotypes, and the impact of MAF of the studied variants was assessed using recessive, dominant, and combined genotype models. Gene-environmental interaction were analyzed using two-by-four tables (Botto and Khoury 2001), and Rothman’s synergy index (S) index was calculated to determine whether there is a relative increase (> 1) or decrease (< 1) in the influence of the two factors studied. Alleles were tested for compatibility with the HWE using a *χ*^*2*^ test. Post hoc power analysis for associations was conducted using the Quanto software. Multivariate analyses were performed using multivariate logistic regression to control for confounding factors.

The meta-analysis utilized the METAGENYO software, available at the following URL: http://bioinfo.genyo.es/metagenyo/ (accessed on December 10, 2023) (Martorell-Marugan et al. 2017). For each variant, six comparisons were conducted: allele contrast (A vs. B), recessive model (AA vs. AB + BB), dominant model (AA + AB vs. BB), overdominant model (AB vs. AA + BB), the effect of homozygotes AA vs. BB, and the effect of heterozygotes AB vs. BB. The Cochran’s *Q* test and *I*^2^ statistic were utilized to assess heterogeneity among the selected studies (Higgins and Thompson 2002). In instances where heterogeneity exceeded 50% (*I*^2^ > 50%), the random effects model (REM) was applied; otherwise (*I*^2^ ≤ 50%), the fixed effects model (FEM) was utilized. For the tested associations, ORs, *P*-values (with 95% CIs), and adjusted *P*-values were computed. Additional tests for HWE were conducted separately for each gene variant among control subjects with both *P*-values and adjusted *P*-values which were calculated for each test. Potential publication bias was evaluated through visual inspection of the funnel plot and Egger’s test.

Results were calculated for both ROP and treatment-requiring ROP. All reported probabilities (*P*-values) were two-sided, with statistical significance defined as *P* < 0.05. STATISTICA version 10.0 and GraphPad Prism version 6.04 were employed for statistical analyses, with the exception of power analysis and meta-analysis.

## Results

### Study population

**Fig. 1:**
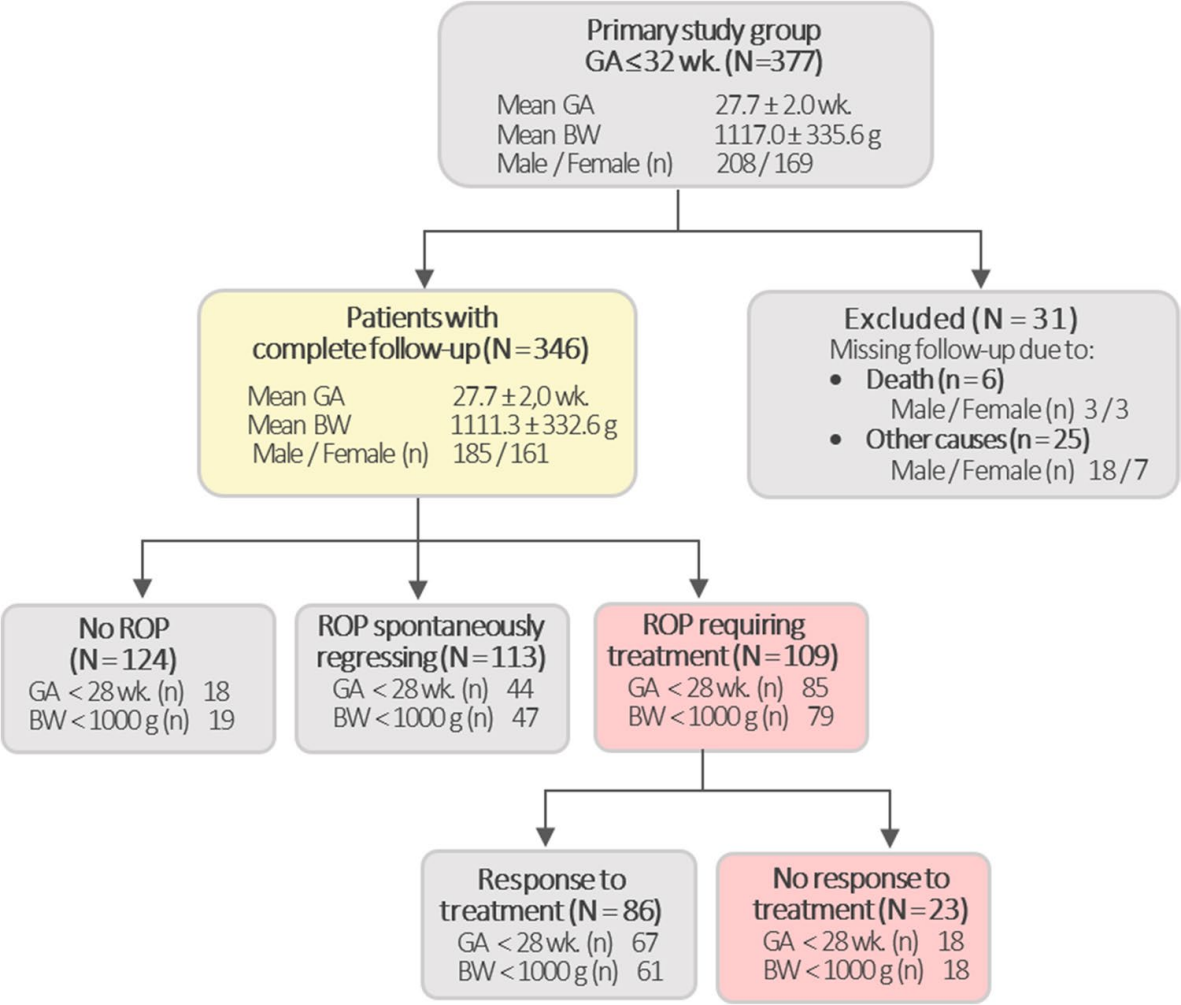
Consolidated scheme of the study’s inclusion criteria and population characteristics

Population characteristics are depicted in Fig. 1. Out of the 377 children comprising the study cohort, a total of 346 individuals (91.7%) completed the entire neonatal and ocular follow-up. Among them, 222 children were with ROP. Within this subgroup, 113 children (50.9%) exhibited ROP that regressed spontaneously, while 109 (49%) had ROP requiring treatment, which included laser photocoagulation (LP) 76 infants (69.7%), intravitreal ranibizumab (IVR) injections 31 infants (28.4%), and a combination of both methods in 2 infants (1.8%). Treatment failure was noted in 23 cases, constituting 21.1% of the group requiring treatment.

### Clinical risk factors of ROP development and unsuccessful treatment

Table 1 provides comprehensive characteristics of the 346 premature infants included in the study, as well as risk factors for developing ROP and treatment failure. Briefly, among this cohort, 185 were male (53.5%), the mean GA of the study group was 27.7 weeks (range: 22–33 weeks), and the mean BW was 1117 g (range: 432–2340 g). The incidence and progression of ROP exhibited an inverse correlation with GA, BW, Apgar scores at 1 min, and 5 min post-birth, as well as parameters associated with respiratory failure, including surfactant treatment, resuscitation, and mechanical ventilation (each of them significant at *P* < 0.0001). Diagnosis of ROP was more prevalent among patients who experienced additional complications of prematurity, including RDS, IVH, DWMI, NEC, and BPD. In the ROP group requiring treatment, no clinical factor was significantly associated with treatment failure.

**Table 1:** Demographic and clinical parameters associated with ROP and failure of its treatment

Parameter	Incidence and outcome of ROP; *N* = 346				ROP requiring treatment; *N* = 109		
	I No ROP *N* = 124	II ROP Not Requiring Treatment *N* = 113	III ROP Requiring Treatment *N* = 109	*p*_*for trend*_ I/II/III	IIIa ROP treated successfully *N* = 86	IIIb ROP treated unsuccessfully *N* = 23	*p* IIIa vs. IIIb
Gestational age [wk.]; mean (SD)	29.1 (1.4)	27.6 (1.7)	26.0 (1.8)	< 0.0001	26.1 (1.8)	25.8 (1.6)	0.875
Gestational age < 28 wk	18 (14.5)	44 (38.9)	85 (88.0)	< 0.0001	67 (77.9)	18 (78.3)	0.769
Body weight [g] mean (SD)	1340.9 (324.5)	1070.4 (264.4)	891.6 (233.9)	< 0.0001	904.8 (247.8)	846.1 (175.1)	0.813
Body weight < 1000 g	19 (15.3)	47 (41.6)	79 (72.5)	< 0.0001	61 (70.9)	18 (78.3)	0.604
Male sex; *n* (%)	63 (50.8)	62 (54.9)	60 (55.0)	0.585	50 (58.14)	10 (43.5)	0.209
Apgar 1; Me (Q1; Q3)	6 (4; 8)	4.5 (2; 6)	3 (2; 6)	< 0.0001	3 (2; 6)	3.5 (1; 6)	0.999
Apgar 5; Me (Q1; Q3)	7 (7; 9)	7 (6; 8)	7 (6; 7)	< 0.0001	7 (6; 7)	7 (6; 7)	0.989
Parameters related to respiratory failure							
Surfactant treatment; *n* (%)	42 (33.9)	48 (42.5)	77 (70.6)	< 0.0001	62 (72.1)	15 (65.2)	0.608
Resuscitation; *n* (%)	86 (69.4)	100 (88.5)	104 (95.4)	< 0.0001	81 (94.2)	23 (100.0)	0.582
Mechanical ventilation; *n* (%)	55 (44.4)	81 (71.7)	104 (95.4)	< 0.0001	81 (94.2)	23 (100.0)	0.582
Mechanical ventilation period [d]; mean (SD)	10.1 (14.7)	21.6 (22.0)	39.5 (28.6)	< 0.0001	40.8 (31.2)	35.7 (18.8)	0.764
Complications of prematurity;* n* (%)							
RDS	75 (60.5)	66 (58.4)	89 (81.7)	0.0008	72 (83.7)	17 (73.9)	0.280
IVH	42 (33.9)	67 (59.3)	93 (85.3)	< 0.0001	71 (82.6)	22 (95.7)	0.187
DWMI	1 (0.8)	12 (10.6)	14 (15.3)	0.0004	12 (14.0)	2 (8.7)	0.503
NEC	15 (12.1)	22 (19.5)	36 (33.0)	0.0002	27 (31.4)	9 (39.1)	0.618
BPD	15 (12.1)	47 (41.6)	77 (70.6)	< 0.0001	61 (70.9)	16 (69.6)	1.000

Abbreviations and symbols: *BPD* bronchopulmonary dysplasia, *DWMI* diffuse white matter injury, *IVH* intraventricular hemorrhage, *NEC* necrotizing enterocolitis, *RDS* respiratory distress syndrome, *ROP* retinopathy of prematurity. Statistical measures: *Mean (SD)*, mean (standard deviation); *Me (Q1; Q3)*, median (interquartile range); n (%), number (percentage)

### *ACE* and *AGTR1* genotypes and ROP occurrence, progression, and treatment failure

Observed overall MAF (minor allele frequency) of the *ACE I* allele in the study cohort was 0.481, and the *AGTR1* rs5186C allele was 0.258. We observed that the frequencies of all studied variants were consistent with HWE (*P*_*HWE*_ > 0.05; Table 2). Univariate analysis revealed an association between the *AGTR1* rs5186C allele and the development of ROP requiring treatment. An increased prevalence of the rs5186C allele in cases with ROP requiring treatment (0.306) as compared with the frequency in infants without ROP (0.260) and those with spontaneously regressing ROP (0.201) was observed. The related increase in the risk of development of treatment—requiring ROP for CC homozygotes—was 2.5-fold (*P* = 0.028), and progression of ROP to advanced stages was 3.0-fold (*P* = 0.032) (Table 2). Both of these effects retained statistically significant after adjusting for GA (1 week increase; *P*_adjusted_ = 0.047 and *P*_adjusted_ = 0.041, respectively), but not when adjusting for the two most pivotal covariates GA and BW < 1000 g (both adjusted *P*-values were above 0.05). A notably elevated frequency of C allele and CC homozygotes was observed in cases where with ROP treatment failed: 0.457 and 26.1%, respectively. For the comparison of genotype frequencies between cases with ROP treatment failure and infants without ROP, the observed odds ratio (OR) was as high as 6.2 with a 95% confidence interval (CI) of 1.71–22.4, and the *P*-value was 0.003. This effect maintained significance after adjusting for both GA and BW < 1000 g (*P*_adjusted_ = 0.028). On the contrary, no statistically significant associations were detected between the studied *ACE I/*D variant and ROP occurrence, progression, and the efficacy of treatment (Table 2).

**Table 2:** Distribution of the studied polymorphism in preterm infants according to the presence, advancement, and the effect of treatment of retinopathy of prematurity (ROP)

Genotype	ROP occurrence and advancement			OR (95%CI); *P*		ROP treatment		OR (95%CI); *P*
	I No ROP *N* = 124	II Non-treatment-requiring ROP *N* = 113	III Treatment-requiring ROP *N* = 109	I + II vs. III	II vs. III	IIIa ROP treated successfully *N* = 86	IIIb ROP treatment failure *N* = 23	IIIa vs. IIIb
*ACE* I/D								
*DD*	32 (26.0)	29 (25.7)	31 (28.4)	1.0	1.0	26 (30.2)	5 (21.7)	1.0
*ID*	62 (50.4)	58 (51.3)	54 (49.5)	0.89 (0.52–1.5); 0.658	0.87 (0.47–1.6); 0.666	42 (48.8)	12 (52.2)	1.5 (0.47–4.7); 0.499
*II*	29 (23.6)	26 (23.0)	24 (22.0)	0.86 (0.45–1.6); 0.646	0.86 (0.41–1.8); 0.702	18 (20.9)	6 (26.1)	1.7 (0.46–6.6); 0.415
*I* allele freq; *P*_*HWE*_	0.488; 0.923	0.487; 0.772	0.468; 0.958	0.93 (0.67–1.3); 0.635	0.93 (0.64–1.3); 0.691	0.453; 0.891	0.522; 0.827	0.76 (0.40–1.46); 0.410
*AGTR1* rs5186								
*AA*	67 (54.5)	73 (65.2)	53 (49.1)	1.0	1.0	45 (52.9)	8 (34.8)	1.0
*AC*	48 (39.2)	33 (29.5)	42 (38.7)	1.4 (0.84–2.2); 0.206	1.75 (0.98–3.1); 0.056	33 (38.8)	9 (39.1)	1.5 (0.54–4.4); 0.424
*CC*	8 (6.5)	6 (5.4)	13 (12.3)	**2.5 (1.1–5.6); 0.028**^**a**^	**3.0 (1.1–8.4); 0.032**^**b**^	7 (8.2)	6 (26.1)	4.8 (1.3–18.1); 0.014^c^
*C* allele freq; *P*_*HWE*_	0.260; 0.988	0.201; 0.306	0.315; 0.306	**1.5 (1.1–2.2); 0.019**	1.8 (1.2–2.8); 0.006	0.276; 0.904	0.457; 0.306	2.2 (1.1–4.3); 0.020

Abbreviations and symbols: *P*_*HWE*_ statistical significance for comparison between observed genotype frequencies and those expected based on Hardy–Weinberg equilibrium. Multivariate statistical analysis: data adjusted for GA (increase by 1 week) and BW < 1000 g: (a) *P*_*adjusted*_ = 0.058 (GA only: *P*_*adjusted*_ = 0.047); (b) *P*_*adjusted*_ = 0.052 (GA only: *P*_*adjusted*_ = 0.041); (c) *P* = 0.028

### GxE interaction between *AGTR1* genotype, ELBW, and ROP requiring treatment

The observed modifying effect of BW on the statistical significance of the *AGTR1* genotype’s effect on ROP risk suggests the existence of a GxE interaction. A significant interaction was observed between *AGTR1* rs5186CC risk genotype and ELBW on the development of advanced ROP. The individual effect of ELBW was found to increase risk 7.1-fold, the rs5186CC genotype 2.1-fold, and the co-occurrence of both factors was linked to 12.4-fold increase in risk (*P* < 0.0001; Supplementary Table S2). The OR expected based on individual effect was only 8.2.

### *ACE* and *AGTR1* genotypes and comorbidities

No significant associations were identified between the examined genetic variants of *ACE* and *AGTR1* and complications of prematurity (Supplementary Table S3). The sole observation was that *AGTR1* rs5186CC homozygotes exhibit a heightened incidence of ELBW (OR was 2.11 (95% CI 0.95–4.69); *P* = 0.063). However, this effect appears to be influenced by the GxE interaction.

### Meta-analysis of the effect of *ACE* and *AGTR1* genotypes on ROP

**Fig. 2:**
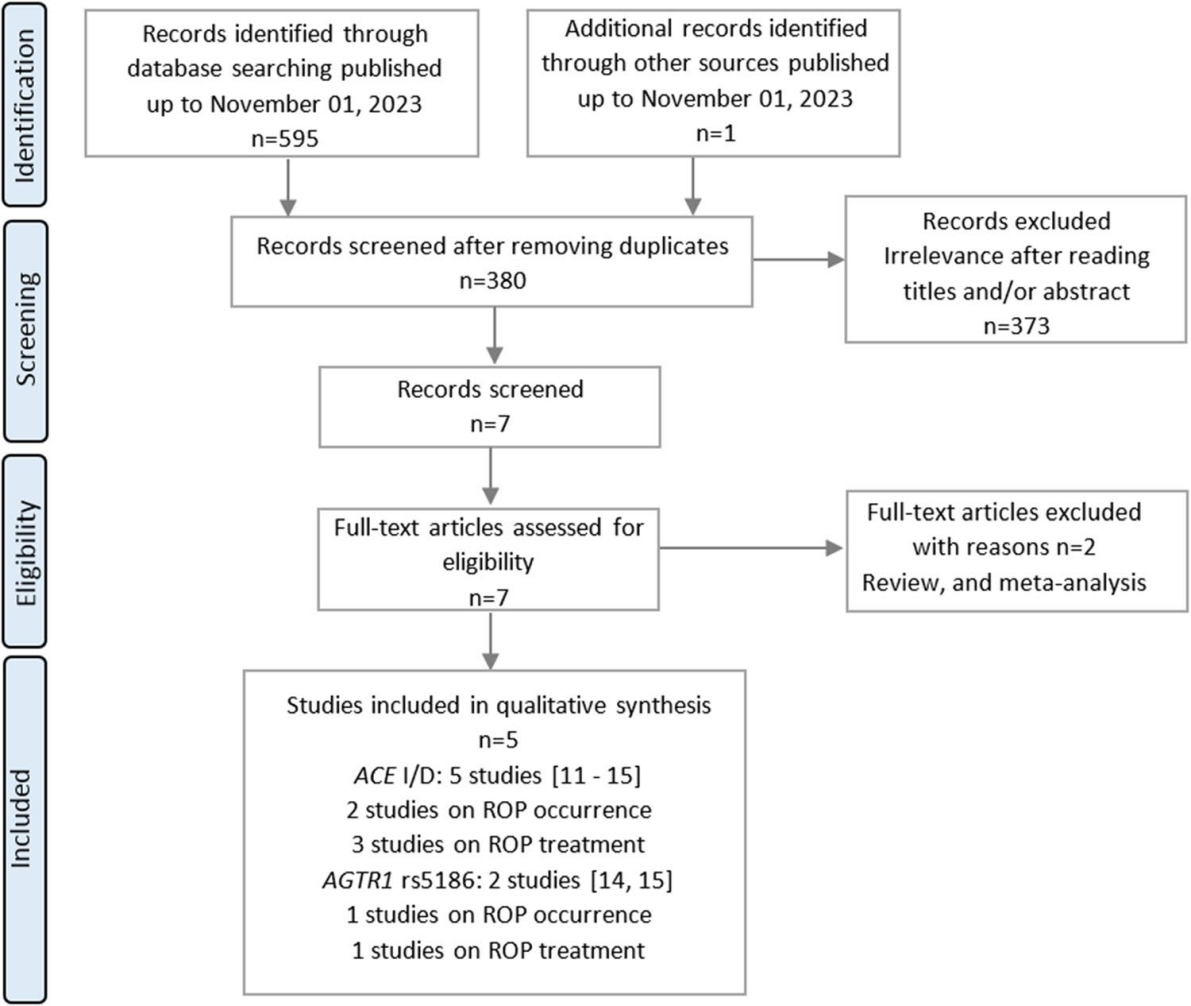
Flow diagram illustrating the selection process of eligible studies for the meta-analysis of the effect of *ACE* and *AGTR1* variants

Our initial search of the databases yielded a total of 595 potentially relevant articles (Fig. 2). After evaluating the titles and abstracts, we excluded 215 duplicate and 373 obviously irrelevant studies. Subsequently, the eligibility of the remaining studies was evaluated, leading to the exclusion of two studies due to invalid data (review or letter to the editor) and overlapping data. Ultimately, a total of 4 case–control (Haider et al. 2002; Yildiz et al. 2010; Lei et al. 2018; Poggi et al. 2015) and 1 cohort (Spiegler et al. 2010) studies comprising 996 cases and 2787 controls in the case of *ACE* and 191 cases and 1661 controls in the case of *AGTR1* were included in the meta-analysis, along with data analyzed in the present study.

**Fig. 3:**
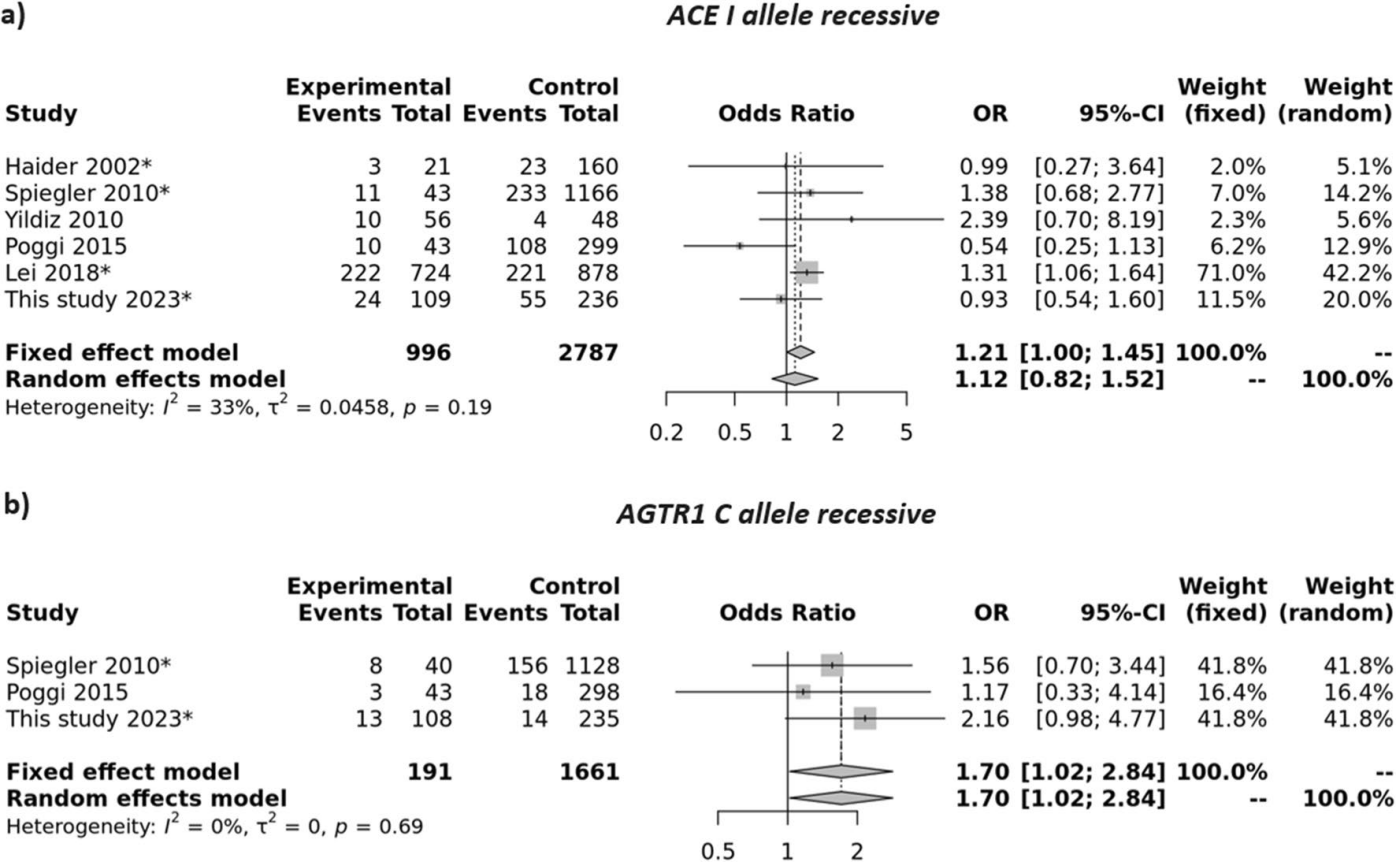
Forest plot on the association of *ACE* I/D (**a**) and *AGTR1* rs5186 (**b**) variant with the development of ROP. Recessive model of inheritance is presented. Studies in which advanced ROP was assessed were marked with an asterisk (*). In the remaining cases, any stage of ROP was assessed

**Fig. 4:**
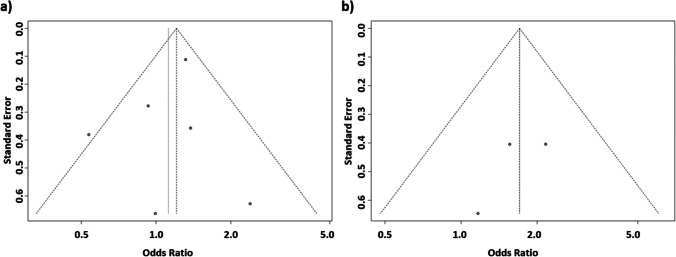
Funnel plot on the association of *ACE* I/D (**a**) and *AGTR1* (**b**) variant with the development of ROP (recessive model of inheritance)

The main characteristics of articles included in the meta-analysis are listed in Table 3. Studies of the *ACE* variant have been conducted across Asian (China, Kuwait, Turkey) and European (Germany, Italy, Poland) regions, but *AGTR1* only in Europe (Germany, Italy, Poland). Based on the quality assessment of NOS, all six studies, including ours (Haider et al. 2002; Yildiz et al. 2010; Lei et al. 2018; Poggi et al. 2015; Spiegler et al. 2010), were of high quality. Notably, adjustments for potential confounding factors varied among studies, and the main factors adjusted were GA, BW, Apgar score, and parameters related to mechanical ventilation or other treatment. The outcomes are presented in Table 4 and depicted in Figs. 3 and 4.

**Table 3:** Characteristics of studies included in the meta-analysis of *ACE rs4646994* and *AGTR1 rs5186* variants

First author and year	Country (Ethnicity)	Method	ROP	Cases/controls	Cases					Controls					WG MAFs	WG *P*_HWE_	Controls *P*_HWE_	Q score
*ACE I/D*					II	ID	DD	I	D	II	ID	II	I	D				
Haider et al. 2002	Kuwait (Asian)	PCR	**Adv**	21/ 160	3	5	13	11	31	23	73	64	119	201	0.359	0.692	0.768	7
Spiegler et al. 2010	Germany (Caucasian)	PCR	**Adv**	43/1166	11	23	9	45	41	233	601	332	1067	1265	0.460	0.400	0.190	7
Yildiz et al. 2010	Turkey (Caucasian)	PCR	Any	56/48	10	23	23	43	69	4	23	21	31	65	0.356	0.938	0.507	7
Poggi et al. 2015	Italy (Caucasian)	PCR	Any	43/299	10	22	11	42	44	108	144	47	360	238	0.588	1.000	0.931	7
Lei et al. 2018	China (Asian)	PCR	**Adv**	724/878	222	341	161	785	663	221	428	229	870	886	0.517	0.298	0.459	9
This study 2024	Poland (Caucasian)	PCR	**Adv**	109/236	24	54	31	102	116	55	120	61	230	242	0.481	0.983	0.787	9
*AGTR1 rs5186*					CC	AC	AA	C	A	CC	AC	AA	C	A				
Spiegler et al. 2010	Germany (Caucasian)	PCR	**Adv**	40/1128	8	17	15	33	47	156	427	545	739	1517	0.330	< 0.0001	0.000	7
Poggi et al. 2015	Italy (Caucasian)	TaqMan	Any	43/298	3	17	23	23	63	18	92	188	128	468	0.221	0.404	0.144	7
This study 2024	Poland (Caucasian)	TaqMan	**Adv**	108/235	13	42	53	68	148	14	81	140	109	361	0.258	0.502	0.618	9

Abbreviations: *Adv*, advanced ROP; *Any*, any stage of ROP; *MAF*, minor allelic frequency; *PCR*, polymorphism chain reaction; *P*_*HWE*_, statistical significance for comparison between observed genotype frequencies and those expected based on Hardy–Weinberg equilibrium; *Q*, quality; *WG*, whole group

**Table 4:** Meta-analysis results of association of *ACE* and *AGTR1* variants with ROP risk

Variant/analysis	Genetic model		Type of model	Heterogeneity		Statistical analysis				Publication bias; *P*_Eggers_
				*I*^2^ (%)	*P*_*H*_	OR	95% CI	*P*_crude_	*P*_adj_	
*ACE* rs4646994	Allele contrast	I vs. D	REM	59	0.033	1.00	0.78; 1.27	0.995	1.000	0.219
	Recessive	II vs. ID + DD	FEM	33	0.191	1.21	1.00; 1.45	0.045	0.314	0.562
	Dominant	II + ID vs. DD	REM	50	0.075	0.94	0.67; 1.32	0.733	1.000	0.169
	Overdominant	ID vs. II + DD	FEM	0	0.583	0.93	0.79; 1.09	0.374	1.000	0.459
	Homozygotes 1	II vs. DD	REM	52	0.063	1.07	0.68; 1.68	0.771	1.000	0.397
	Homozygotes 2	II vs. ID	FEM	3	0.395	1.19	0.98; 1.45	0.079	0.555	0.949
	Heterozygotes	ID vs. DD	REM	28	0.228	1.02	0.84; 1.24	0.856	1.000	0.152
*AGTR1* rs5186	Allele contrast	C vs. A	FEM	0	0.919	1.45	1.14; 1.86	0.003	0.021	0.135
	Recessive	CC vs. AC + AA	FEM	0	0.692	1.70	1.02; 2.84	0.042	0.291	0.463
	Dominant	CC + AC vs. AA	FEM	0	0.995	1.53	1.10; 2.11	0.011	0.074	0.917
	Overdominant	AC vs. CC + AA	FEM	0	0.887	1.27	0.91; 1.76	0.154	1.000	0.560
	Homozygotes 1	CC vs. AA	FEM	0	0.741	1.99	1.16; 3.43	0.013	0.091	0.297
	Heterozygotes 2	CC vs. AC	FEM	0	0.675	1.39	0.81; 2.4	0.235	1.000	0.385
	Heterozygotes	AC vs. AA	REM	0	0.973	1.42	1.01; 2.01	0.045	0.314	0.309

Abbreviations: *FEM* the fixed effects model, *REM* the random effects model

As illustrated in Table 4 and Fig. 3, our meta-analysis substantiates the involvement of the *ACE* and *AGTR1* variants in the pathogenesis of ROP. The analysis considered the advanced proliferative form of ROP whenever data were available; otherwise, data from any stage of ROP were included. The overall OR calculated from the six studies indicates a correlation between the presence of the *ACE* II genotype and the risk of ROP requiring treatment (fixed effects model [FEM]: OR = 1.21, 95% CI: 1.00 – 1.45, *P*_crude_ = 0.045; *P*_adjusted_ = 0.314). There was no statistically significant heterogeneity observed among the included studies (*I*^2^ = 33%, *P*_*H*_ = 0.19).

The funnel plot representing this association displayed a symmetrical pattern (Fig. 4a), indicating the absence of publication bias in the studies. This observation was further supported by Egger’s test (*P* = 0.562). For *AGTR1*, the analysis revealed an overall OR calculated from three studies, indicating positive correlation between the presence of the rs5186CC genotype and the risk of ROP requiring treatment (fixed effects model [FEM]: OR = 1.70, 95% CI 1.02–2.84, *P*_crude_ = 0.042; *P*_adjusted_ = 0.291). No statistically significant heterogeneity was observed among the included studies (*I*^2^ = 0%, P_H_ = 0.69). The funnel plot representing this association displayed a symmetrical distribution (Fig. 4b), suggesting an absence of publication bias in the studies. This observation was corroborated by Egger’s test (*P* = 0.463).

## Discussion

ROP stands as a primary cause of childhood blindness, with risk factors for the ROP progression which are based upon GA (≤ 30 weeks) and BW (≤ 1500 g) (Broxterman and Hug 2016). Effective early treatment methods such cryotherapy, laser photocoagulation, and anti-vascular endothelial growth factor (VEGF) medication can prevent further visual loss by retinal detachment; however, due to the rapid progression of ROP, early detection is critical.

Despite improvements in obstetric and neonatal care in developed European countries, the incidence of advanced ROP remains a significant clinical problem, as it is observed that with a constant incidence of ROP, the number of patients requiring treatment appears to be increasing. In a study conducted at our Gynecology and Obstetrics Clinical Hospital in Poznan from 2017 to 2019, an average of 443 infants were screened for ROP each year, with 26% developing any stage of ROP and 10% (approximately 45 patients per year) developing the proliferative type (Chmielarz-Czarnocińska et al. 2021). A previous study in Sweden observed that 30% of infants developed ROP and 5.2% developed the proliferative type, with no change in treatment frequency over a 5-year period (Holmström et al. 2016). In Denmark, the incidence of treated ROP cases significantly increased from 1.3% during 1996–2000 to 3.5% during 2001–2005 (Slidsborg et al. 2008). It is indicated that the observed increase in the number of treated cases cannot be fully explained by increased neonatal survival rates or changes in neonatal risk factors.

In this study, a significant and specific association between the *AGTR1* rs5186C allele and the advancement of ROP and the effects of its treatment was established. The rs5186CC homozygotes had a 2.47-fold increased risk of developing a proliferative, treatment-requiring type of ROP (*P* = 0.027), along with a 4.82-fold risk of treatment failure (*P* = 0.014). Notably, the significance of this genotype may be magnified by genetic-environmental interactions, particularly in cases of coexisting ELBW. Meta-analysis confirm the role of rs5186C allele in ROP. Despite the fact that ACE is considered an essential factor because of its developmental function and that prior studies showed substantial results in a decreased risk of ROP, there was no association between studied *ACE* I/D variant and ROP in the Polish population. However, meta-analysis insights suggest that I allele may potentially serve as a risk factor for ROP. No associations were found between *ACE* and *AGTR1* and other complications of prematurity.

Vascular development in the retina is not completed until 40 weeks of GA. In utero, physiologic hypoxia and serum insulin-like growth factor-1 (IGF1), which regulate VEGF levels, were driving retina vessels subsequent development (Hård et al. 2013). Following birth, preterm infants, due to a hyperoxia and relative nutritional deficiency, experience lower serum IGF1 levels, which result in delayed retinal vascularization (Hård et al. 2013). The progress of ROP, which leads to total retinal detachment, initiates with the obliteration of developing capillaries. Subsequently, pathologic angiogenesis predominates over normal development, leading to the growth of extraretinal fibrovascular proliferation or neovascularization into the vitreous (Das and Byrd 2014). RAAS genes have been implicated in normal and pathological angiogenesis, including their interaction with IGF pathways, which contributes to the development of diabetic retinopathy (Lovshin et al. 2019). However, studies investigating these pathways’ genes in the context of neonatal diseases remain limited. There is particularly paucity of data on *AGTR1*, with analyses predominantly confined to Caucasian populations.

The association between the *AGTR1* genotype and the ROP was first demonstrated by Mohamed et al. (2009). The study encompassed 347 infants less than 32 weeks GA, including 92 ROP cases. They found the robust association between intragenic SNP *AGTR1* rs427832 at *P* = 0.005. However, further studies, by Poggi et al. (2015), failed to confirm the significant role of *AGTR1* in ROP development. In their retrospective analysis involving 342 preterm neonates with a GA < 28 weeks, including 43 cases, the risk of ROP development was on the significance level of tendency for *rs5186C* allele carriers (OR = 1.47; *P* = 0.238, Table 3). Comparing these findings with our own, a joint analysis revealed a 1.27-fold increased risk of ROP development for *rs5186C* allele carriers, although this remained statistically insignificant (*P* = 0.144). In a more recent investigation by Rathi et al. (2017), *AGTR1* rs2739504 intronic variant (g.13100A > G) was identified as a risk factor for ROP (OR = 1.36 95%CI 1.01–1.83; *P* = 0.041). Their study involved a comprehensive screening of candidate genes in 189 preterm infants with ROP and 167 no-ROP to identify variants conferring susceptibility to the disease. 16 SNPs in *AGTR1* were analyzed, revealing an observed allele frequency of 0.446 in cases and 0.371 in controls.

The rs5186 variant in the *AGTR1* gene can affect protein mRNA stability and translation (Mottl et al. 2008; Figueroa et al. 2023). This variant targets gene-expression-regulating miR155. Experimental studies involving reporter silencing assays have demonstrated that miR155 downregulates the expression of the 1166A allele of rs5186 but not the 1166C allele (Sethupathy et al. 2007), thereby associating the C allele is with gene upregulation (Musso et al. 2019). This gain-of-function variant has been linked to various health issues, including hypertension, cardiovascular disease, and metabolic syndrome. It also affects the renovascular system, promoting tissue lipid accumulation, inflammation, and organ damage (Musso et al. 2019). Furthermore, the rs5186AC + CC genotype adversely affects endothelial functions, leading to vascular remodelling and insulin resistance (de Gracia Hahn et al. 2019; Potaskalova et al. 2022; de Gracia Hahn et al. 2019).

Miller et al. (1999) provided that the rs5186C allele variant is related to augmented Ang II activity. In vitro studies have shown that Ang II promotes angiogenesis (Hu et al. 2007; Le Noble et al. 1991). Moreover, the experimental rat models have highlighted a significant role for the Ang II-AGTR1 interactions during postnatal kidney development**—**Ang II induces capillary endothelial expansion and the formation of the outer medullary vascular bundle, as well as Ang II-mediated stimulation of VEGF release from the epithelium (Madsen et al. 2010). Ang II generates oxidative stress in the vasculature, leading to endothelial dysfunction. This stress initiates cellular pathways, causing endothelial cell apoptosis. Ang II senescence reduces endothelial cells regenerative capacity and increases thrombogenicity due to increased adhesion molecule expression (St Paul et al. 2020).

There are a number of pharmacological treatment options developed to inhibit RAAS in cardiovascular and renal diseases, which can also be explored in experimental and clinical studies on ROP (Ksiazek et al. 2024). The present study underscores the potential significance of topical AGTR1 receptor inhibition as a viable treatment option for ROP.

### Limitations

The first limitation is the lost from follow-up 31 cases including 6 due to death. However, the number of these cases does not exceed 10% of the entire population, and there are negative results of associations between studied variants and death of premature newborns (Spiegler et al. 2010). The second limitation is sample size; post hoc statistical power analysis for significant associations in the Polish population study, conducted using Quanto software, revealed moderate statistical power for analysing recessive effects of the *AGTR1* C allele. The power was approximately 70% for both the incidence of ROP and the progression of ROP to advanced stages and 63% for the *AGTR1*-ELGW interaction. However, the advantages of our research include the assessment of a large group of infants with ROP and the identification of a group of those requiring treatment. The third limitation of this study is the limited number of population included in meta-analysis. For the *ACE* variant, there were no cases from Americas, while for *AGTR1* only, European Caucasian populations were available. Therefore, the interpretation of the results, especially for *AGTR1* variant, may be restricted to specific populations and should be replicated in other cohorts. The fourth limitation is that the results primarily reflects pre-COVID-19 pandemic data. The observed effects of the RAS gene variants should be cautiously replicated post-pandemic, as information from specific countries on the impact of lockdown period (resulting in delayed screening) on the risk of ROP development and progression (Chakraborty and Sheth 2023) from one said and the possible impact of these variants on the health of a newborn from the other. These variants were found to modify the severity of symptoms associated with SARS-CoV-2 infection and the outcome of COVID-19 (Martinez-Fierro et al. 2024).

## Conclusions

Evidence indicates that the *AGTR1* gain-of-function variant, due to its role in promotion of angiogenesis and pro-inflammatory effects, may have a significant impact on ROP development. Screening for this variant could aid in identifying premature newborns at risk of progressing to advanced stages of ROP. Developing personalized treatment strategies that incorporate the local use of RAAS inhibitors may prove beneficial in preventing the adverse outcomes of ROP.

### What’s new?

The renin–angiotensin–aldosterone system (RAAS) is crucial for maintaining vital organ perfusion and plays a role in proper vessel development as well as pathological angiogenesis. Growing evidence links RAAS genes, including *ACE* and *AGTR1*, to retinopathy of prematurity (ROP). An association study conducted in the Polish population, along with a meta-analysis, confirmed the impact of the *AGTR1* gain-of-function variant on ROP in Caucasian populations. Noteworthy aspects of research include the examination of a large ROP infant cohort from the Polish population and the identification of those needing treatment. Particularly, the frequency of *AGTR1* rs5186CC homozygotes was observed to be 12% in the treated group compared with 5.8% in other infants studied.

## Supplementary Information

Below is the link to the electronic supplementary material.

Supplementary file1 (DOCX 26 KB)

## Data Availability

All data generated or analyzed during this study are included in this published article and its supplementary information files. Additional data that support the findings of this study are available from the corresponding author upon reasonable request.
