# Supplementary material for: Association of ACE and AGTR1 variants with retinopathy of prematurity: a case–control study and meta-analysis
Source: J Appl Genet. 2024 Aug 26;66(4):911–23. doi: 10.1007/s13353-024-00900-0 (PMC12605436; doi:10.1007/s13353-024-00900-0)
Supplement: Supplementary file 1 — Supplementary file1 (DOCX 26 KB) [file 13353_2024_900_MOESM1_ESM.docx]

**Supplementary Material**

**Title**: Association of *ACE* and *AGTR1* variants with retinopathy of prematurity: a case-control study and meta-analysis

**Authors**: Anna Durska^1^, Dawid Szpecht^2^, Anna Gotz-Więckowska^3^, Ewa Strauss^1^

1 Institute of Human Genetics, Polish Academy of Sciences, Strzeszynska 32, 60-479 Poznan, Poland; strauss@man.poznan.pl

2 Chair and Department of Neonatology, Poznan University of Medical Sciences,

3 Chair and Department of Ophthalmology, Poznan University of Medical Sciences, Poznan, Poland;

**Correspondence to:** Ewa Strauss, PhD, Institute of Human Genetics, Polish Academy of Sciences, Strzeszynska 32, 60-479 Poznan, Poland; phone: +48 (61)6579268; strauss@man.poznan.pl

Supplementary Table S1. Methodology for evaluating the studied *ACE* and *AGTR1* variants.

| *ACE* alleles | Primer | | Sequence | | |
| --- | --- | --- | --- | --- | --- |
| I and D^a^ | Forward | | 5’-CTGGAGACCACTCCCATCCTTTCT-3’ | | |
|  | Reverse | | 5’-GATGTGGCCATCACATTCGTCAGAT-3’ | | |
| I^b^ | Forward | | 5’-TGGGACCACAGCGCCCGCCACTAC-3’ | | |
|  | Reverse | | 5’-TCGCCAGCCCTCCCATGCCCATAA-3’ | | |
| PCR conditions | | | | | |
| Steps | | | Temperature (°C) | Duration | Cycles |
| Initial Denaturation | | | 94 °C | 5 min | 1 |
| Denaturation | | | 92 °C | 60 s | 35 |
| Annealing | | | 58 °C^a^ / 67°C^b^ | 60 s |  |
| Extension | | | 72 °C | 2 min 30 s |  |
| Final extention | | | 72 °C | 7 min | 1 |
| *AGTR1* alleles | Fluorescent marker of the probe | | Context sequence | | |
| A | VIC | TGCAGCACTTCACTACCAAATGAGC[A]  TTAGCTACTTTTCAGAATTGAAGGA | | | |
| C | FAM | TGCAGCACTTCACTACCAAATGAGC[C]  TTAGCTACTTTTCAGAATTGAAGGA | | | |
| qPCR conditions | | | | | |
| Steps | | | Temperature (°C) | Duration | Cycles |
| Initial Denaturation | | | 95 °C | 12 min | 1 |
| Denaturation | | | 95 °C | 20 s | 1 |
| Annealing/Extension | | | 60 °C | 60 s | 40 |

a, b-difference in annealing temperature

Supplementary Table S2. Cumulative effect of *AGTR1* rs5186 SNV and ELBW on the presence of ROP requiring treatment, indicating a gene x environmental interaction.

| *AGTR1 rs5186* | BW < 1000 g | ROP I+II  N=235 | ROP III  N=108 | OR (95%CI); *P* |
| --- | --- | --- | --- | --- |
| AA+AC | No | 161 (68.5) | 26 (24.1) | 1.0 |
| AA+AC | Yes | 60 (25.5) | 69 (63.9) | 7.1 (0.15-12.2); <0.0001 |
| CC | No | 9 (3.8) | 3 (2.8) | 2.1 (0.52-8.1); 0.388 |
| CC | Yes | 5 (2.1) | 10 (9.3) | 12.4 (3.9-39.2); <0.0001 |
| OR expected from individual influence | | | | 8.2 < 12.4; *P* <0.0001 |
| The Rothman's synergy index (*S*) | | | | 1.6  relative increase in the effect |

Abbreviations and symbols: BW - birth weight.

Supplementary Table S3. Distribution of the studied polymorphism in preterm infants according to the presence of complications of prematurity: RDS, IVH, BPD, NEC, ELGA (GA< 28 w.) and ELBW (BW< 1000 g).

| **Genotype**  **n (%)** | **RDS** | | **IVH** | | **DWMI** | | **NEC** | | **BPD** | | **GA** | | **BW** | |
| --- | --- | --- | --- | --- | --- | --- | --- | --- | --- | --- | --- | --- | --- | --- |
|  | **No**  **N=116** | **Yes**  **N=230** | **No**  **N=144** | **Yes**  **N=202** | **No**  **N=319** | **Yes**  **N=27** | **No**  **N=273** | **Yes**  **N=73** | **No**  **N=207** | **Yes**  **N=139** | **≥ 28 w.**  **N=199** | **< 28 w.**  **N=147** | **≥ 1000 g**  **N=201** | **< 1000 g**  **N=145** |
| *ACE I/D* | | | | | | | | | | | | | | |
| *II* | 28 (24.1) | 64 (27.9) | 40 (28.0) | 52 (25.7) | 85 (26.6) | 7 (26.9) | 68 (25.0) | 24 (32.9) | 57 (27.5) | 35 (25.4) | 48 (24.2) | 44 (29.9) | 55 (27.5) | 37 (25.5) |
| *ID* | 55 (47.4) | 119 (52.0) | 65 (45.5) | 109 (54.0) | 164 (51.4) | 10 (38.5) | 141 (51.8) | 33 (45.2) | 100 (48.3) | 74 (53.6) | 103 (52.0) | 71 (48.3) | 101 (50.5) | 73 (50.3) |
| *DD* | 33 (28.4) | 46 (20.1) | 38 (26.6) | 41 (20.3) | 70 (21.9) | 9 (34.6) | 63 (23.2) | 16 (21.9) | 50 (24.2) | 29 (21.0) | 47 (23.7) | 32 (21.8) | 44 (22.0) | 35 (24.1) |
| MAF | 0.522 | 0.461 | 0.493 | 0.473 | 0.476 | 0.538 | 0.491 | 0.445 | 0.483 | 0.478 | 0.497 | 0.459 | 0.473 | 0.493 |
| *P* | 0.214 | | 0.247 | | 0.289 | | 0.391 | | 0.617 | | 0.497 | | 0.864 | |
| *AGTR1 rs5186* | | | | | | | | | | | | | | |
| *AA* | 61 (54.0) | 132 (57.4) | 79 (56.0) | 114 (56.4) | 179 (56.6) | 14 (51.9) | 153 (56.7) | 40 (54.8) | 117 (57.1) | 76 (55.1) | 110 (55.8) | 83 (56.8) | 114 (57.6) | 79 (54.5) |
| *AC* | 46 (40.7) | 77 (33.5) | 51 (36.2) | 72 (35.6) | 110 (34.8) | 13 (48.1) | 96 (35.6) | 27 (37.0) | 72 (35.1) | 51 (37.0) | 75 (38.1) | 48 (32.9) | 73 (36.9) | 50 (34.5) |
| *CC* | 6 (5.3) | 21 (9.1) | 11 (7.8) | 16 (7.9) | 27 (8.5) | 0 (0.0) | 21 (7.8) | 6 (8.2) | 16 (7.8) | 11 (8.0) | 12 (6.1) | 15 (10.3) | **11 (5.6)** | **16 (11.0)^a^** |
| MAF | 0.257 | 0.259 | 0.259 | 0.257 | 0.259 | 0.241 | 0.256 | 0.267 | 0.254 | 0.264 | 0.251 | 0.267 | 0.240 | 0.283 |
| *P* | 0.264 | | 0.995 | | 0.162 | | 0.959 | | 0.933 | | 0.285 | | 0.177 | |

Abbreviations and symbols: BW - birth weight; BPD—bronchopulmonary dysplasia; DWMI—diffuse white matter injury; ELBW - extremely low birth weight; GA - gestational age; IVH—intraventricular hemorrhage; NEC—necrotizing enterocolitis; RDS—respiratory distress syndrome; MAF - minor allele frequency. Statistical measures: n (%), number (percentage);

Statistical analysis: BW and *AGTR1* rs5186CC homozygotes (recessive model): OR=2.11, 95%CI (0.95 – 4.69); *P*= 0.063
